# Liver transplantation for spalt-like transcription factor 4-positive dual-phenotype hepatocellular carcinoma with microscopic bile duct tumor thrombus: a case report

**DOI:** 10.1055/a-2646-1513

**Published:** 2025-07-25

**Authors:** Songming Ding, Xiangyan Liu, Zhuoyi Wang, Shanjie Dong, Qinfen Xie, Shusen Zheng, Qiyong Li

**Affiliations:** 1 Division of Hepatobiliary and Pancreatic Surgery, Shulan (Hangzhou) Hospital Affiliated to Zhejiang Shuren University, Shulan International Medical College, Hangzhou, China

Hepatocellular carcinomas (HCCs) with bile duct tumor thrombi (BDTT) have poorer prognoses than those without BDTT 1 . The role of liver transplantation in patients with HCC with BDTT remains controversial 2 3 . Here, we present a patient without macroscopic BDTT before liver transplantation who developed massive BDTT 2 months after liver transplantation.

A 54-year-old man was diagnosed with HCC (4.6 cm in size) using enhanced computer tomography (CT) and magnetic resonance imaging ( Fig. 1 ). The alpha-fetoprotein level was elevated (1864.1 ng/mL). However, due to a portal vein tumor thrombus and a history of hepatitis B-related liver failure, he did not undergo curative surgery and was waiting for a liver transplantation.

**Fig. 1:**
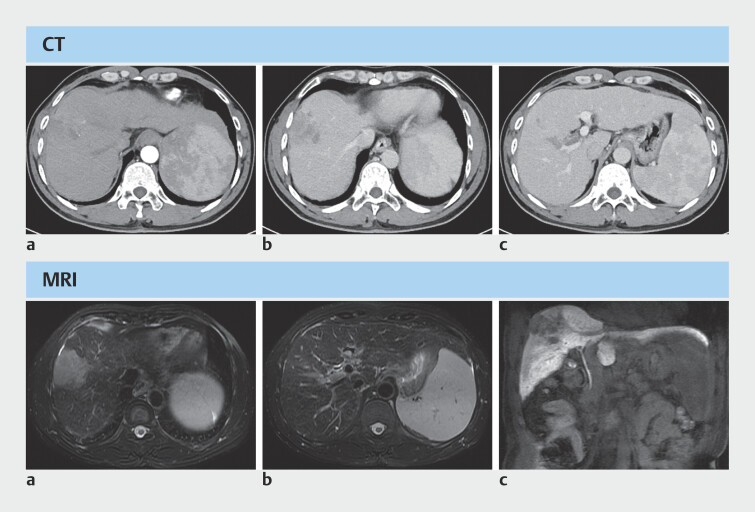
Enhanced computed tomography (CT) and magnetic resonance imaging (MRI) at the patient’s first admission. CT images: **a** arterial phase; **b** venous phase; **c** portal vein tumor thrombus (PVTT) in the right anterior branch and main trunk. MRI images: **a** occupation of liver segment VIII; **b** PVTT in the right anterior branch; **c** no bile duct stricture.

Before liver transplantation, he underwent endoscopic drainage because his CT scan and magnetic resonance cholangiopancreatography revealed a hilar bile duct stricture ( Fig. 2 ). Ten days later, an ABO-compatible deceased donor liver transplantation was performed due to liver failure. Postoperative pathology suggested spalt-like transcription factor 4 (SALL4)-positive dual-phenotype HCC (DPHCC) with negative bile duct margins ( Fig. 2 ). His immunosuppressive regimen was tacrolimus combined with mycophenolate mofetil. During the recovery period, the patient underwent two endoscopic treatments for bile leakage ( Fig. 3 ).

**Fig. 2:**
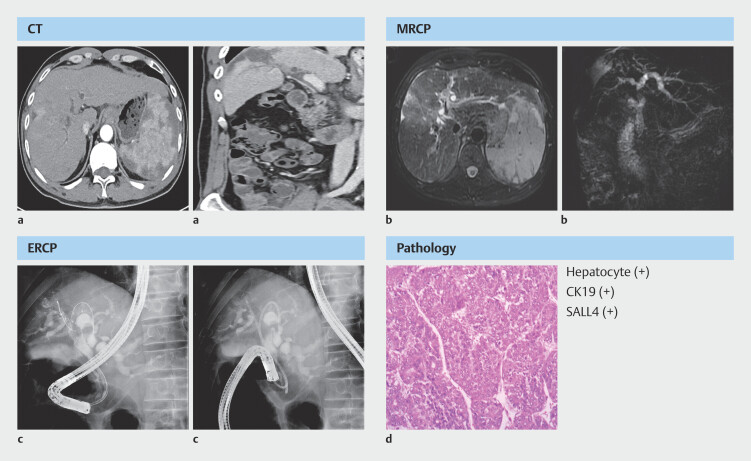
Imaging before liver transplantation. **a, b** Enhanced computed tomography (CT) and magnetic resonance cholangiopancreatography (MRCP) showed hilar bile duct stricture without macroscopic bile duct tumor thrombus. **c** Endoscopic retrograde cholangiography (ERC) drainage. **d** Postoperative pathology of the diseased liver.

**Fig. 3:**
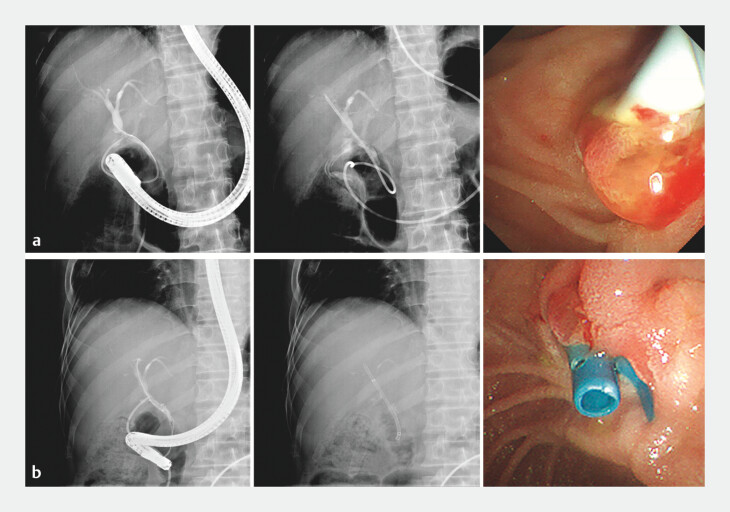
Endoscopic retrograde cholangiography for bile leakage after liver transplantation. **a** Endoscopic nasobiliary drainage. **b** Plastic biliary stent insertion.

Two months after liver transplantation, he developed a rapidly growing BDTT ( Fig. 4 ). Endoscopic retrograde cholangiography and single-operator cholangioscopy using the EyeMax system (Micro-Tech, Nanjing, China) confirmed the infiltrative BDTT ( Video 1 ). The BDTT extended from the lower end of the common bile duct to the hilar bile duct ( Fig. 5 ). Pathological examination of the BDTT revealed SALL4-positive DPHCC ( Fig. 5 ).

**Fig. 4:**
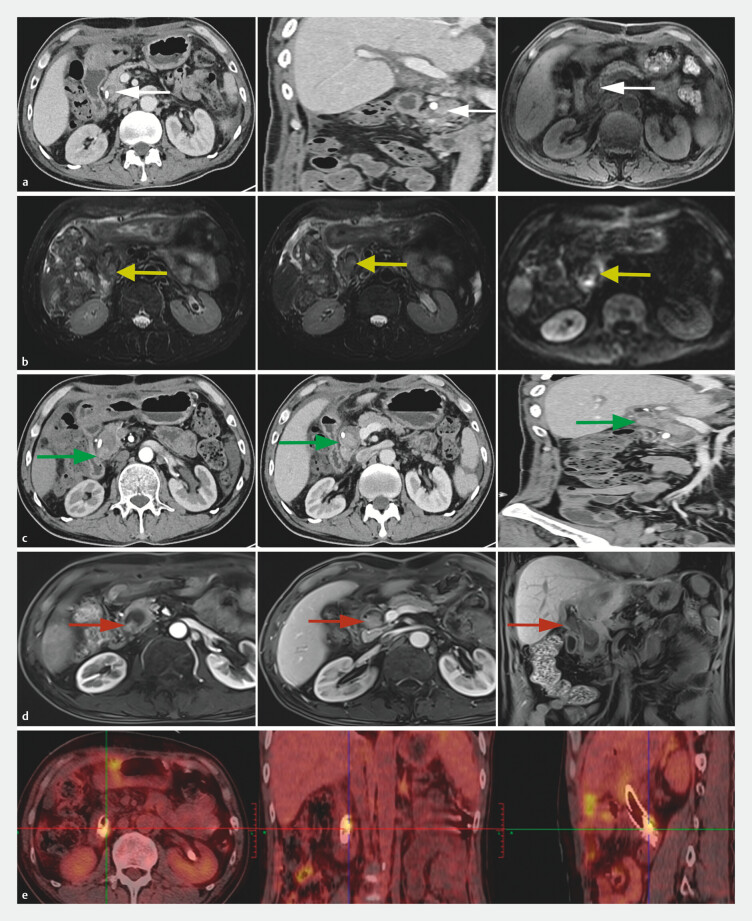
A bile duct tumor thrombus (BDTT) was present and prominent (arrows). **a** Enhanced computed tomography (CT) and magnetic resonance cholangiopancreatography (MRCP) showed a soft tissue shadow in the common bile duct (CBD) 2 months after liver transplantation. **b** MRCP showed a soft tissue shadow filling the CBD 3 months after liver transplantation. **c, d** On CT and magnetic resonance imaging (MRI), the BDTT seemed to adhere tightly to the lower end of the CBD and spread to the hilum of the bile duct. **e** A whole-body positron emission tomography/CT scan showed the BDTT without intrahepatic hepatocellular carcinoma recurrence or metastasis.

Endoscopic retrograde cholangiography and direct view of peroral single-operator cholangioscopy.Video 1

**Fig. 5:**
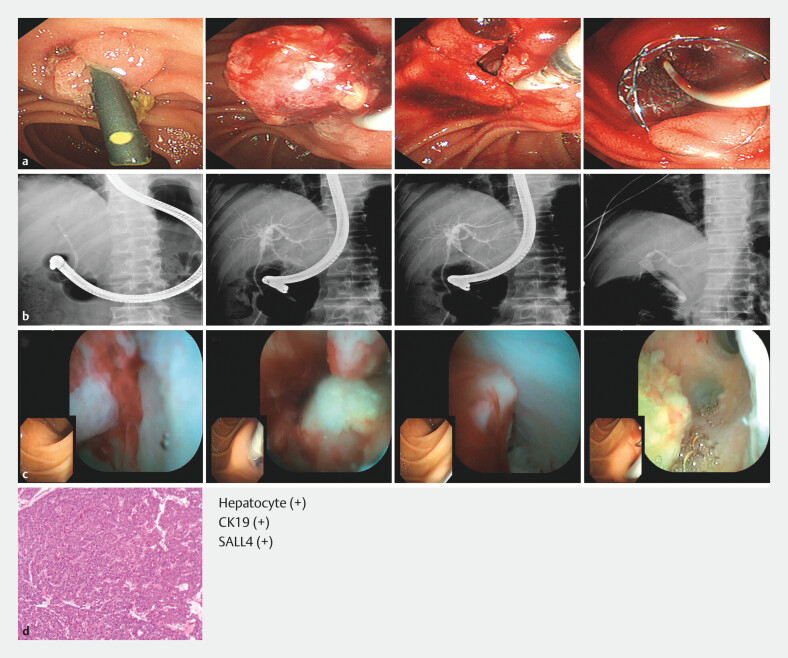
Removal and analysis of the bile duct tumor thrombus (BDTT). **a, b** An endoscopic procedure was performed to remove the BDTT and place a fully covered self-expandable metal stent in the common bile duct (CBD) 3 months after liver transplantation. **c** Single-operator cholangioscopy using the EyeMax system (Micro-Tech, Nanjing, China) showed the BDTT spreading within the CBD 4 months after liver transplantation. **d** Pathological examination of the BDTT suggested that the BDTT was homologous to the primary hepatocellular carcinoma.

The massive BDTT may have had one of the following causes. First, SALL4 is associated with tumorigenesis and is a marker of aggressive HCC 4 , and cytokeratin 19-positive DPHCC is highly invasive 5 . Second, an endoscopic procedure before liver transplantation can cause cancer cell shedding and implantation into the bile duct. Third, using tacrolimus may have caused the massive BDTT.

In conclusion, microscopic BDTT that may cause macroscopic BDTT after liver transplantation should be considered in cases of HCC involving the bile duct tree.

Endoscopy_UCTN_Code_TTT_1AR_2AD

## Notes

Endoscopy E-Videos https://eref.thieme.de/e-videos
*E-Videos* is an open access online section of the journal *Endoscopy* , reporting on interesting cases and new techniques in gastroenterological endoscopy. All papers include a high-quality video and are published with a Creative Commons CC-BY license. Endoscopy E-Videos qualify for HINARI discounts and waivers and eligibility is automatically checked during the submission process. We grant 100% waivers to articles whose corresponding authors are based in Group A countries and 50% waivers to those who are based in Group B countries as classified by Research4Life (see: https://www.research4life.org/access/eligibility/ ). This section has its own submission website at https://mc.manuscriptcentral.com/e-videos .
